# On Union by the First Intention, and Purulent Absorption

**Published:** 1860-05

**Authors:** Warren Stone

**Affiliations:** Professor of Surgery in the University of Louisiana


### ON UNION BY THE FIRST INTENTION AND PURULENT ABSORPTION.

BY WARREN STONE, M. D., PROFESSOR OF SURGERY IN THE UNIVERSITY OF LOUISIANA.

The tendency of us all is to fall into a routine, and to adopt some mode of practice, and to apply it to all cases and conditions; and hence the opposite systems of practice, often seen, not only in different cotntries, but in the same country and in the same city, and even in the same hospital. This is strikingly illustrated by the exclusive and opposite methods of dressing wounds, adopted by the American and French surgeons. The American sugeons dress with a view of obtaining union by the first intention in all cases, whether it is probable it may be effected or not. The French surgeons fill their wounds with lint to prevent union, if there should be a disposition to it. The French, however, I believe, are not so exclusive in their mode of dressing as the Americans, but both are undoubtedly wrong. It is undoubtedly true that our mode of dressing is far better adapted to our country than to France, but it is evident that neither method is adapted exclusively to either country. The American surgeon dresses all cases alike, whether there is prospect of immediate union or not. The wounded or divided parts are brought in close contact, regardless of the consequence of confining dead animal fluids upon surfaces not protected by the exudation of plastic lymph. The French surgeon, fearing purulent absorption of other impure animal matter, leaves the wound open, or fills it with charpie, to absorb any fluid that may accumulate, and to stimulate the surfaces. It is quite certain that, in the French hospitals, at least, adhesive inflamation does not take place so favorably as it does in ours, and I will not pretend to determine how far they are wrong in their exclusive method of dressing; but, favorable as our country is, I am sure that we are as much in error in our exclusive method as they are in theirs. We have not attached sufficient importance to the danger of confining by dressing the effused fluids in the depths of wounds that do not take on healthy, adhesive inflamation. Even if absorption does not take place and affect the system generally, the local effect is bad, causing unhealthy action, and in some cases favoring gangrene. This is shown by the unhealthy inflammation that often follows contused and lacerated wounds, where the blood is diffused in the tissues and the air has sufficient access to favor decomposition.

Hunter, in his work on the blood, warns us against making an opening to discharge the blood that may be extravasated in the tissues after contused wounds, but without giving any other reason, except the fact that such opening is liable to be followed by illconditioned inflammation. While blood is excluded from the air, it remains harmless, and plastic lymph will be exuded around it; but if the air has access to it, decomposition may change it to poison, which will change the character of the inflammation entirely, and reduce the blood to the capacity of the absorbents, and it may be absorbed, and produce more or less constitutional disturbance. I am sure this takes place, under certain circumstances, from blood extravasated into the large serous cavities, for I have seen cases of death, with all the symptoms of animal poisoning, shown by the experiments of Magendie, when the consequent inflamation was very little. This should furnish a plain indication in the management of wounds of this kind; and in the dressing of wounds generally, we should be cautious in confining the animal juices or effusions.

When union by the first intention cannot be expected, it is, at best, useless to bring the surface of the wounds closely together, and even if no other serious consequence follows, the wound itself often takes on unhealthy inflammation, and does not granulate as well as if it had been left more open for the ready discharge of any effused fluid, and for the application of stimulants, if necessary.

It is a great object, in many cases, to effect union by the first intention, in large wounds such as are left after amputation, for the diminished irritation and the saving of the drain of suppuration may often be the means of saving the life of the patient. Something may be done before dressing to lessen the effusion that always takes place from exposed surfaces, and to favor healthy reaction. The effusion of serum would cease, of course, if we waited long enough before dressing, but this is not generally done, and, indeed, in many cases it would require an hour's exposure. The application of ice answers the double purpose, that of drying the stump and affording healthy reaction and the adhesive process.—This, I believe, is a perfectly safe application (unless rigors are produced by it,) and when the capilary circulation is feeble, or when it is feared that too little reaction will take place, there is no other stimulant so wholesome, or that produces so natural and healthy a reaction.

In the French hospitals, purulent absorption is the great dread, and this has led to a more general use of the ecraseur than would otherwise have been given it, for it is asserted (and I believe with truth) that wounds made by this instrument heal more favorably than those made with the knife, and are much less liable to what is termed in Paris purulent absorption. I have reference only to the Paris hospitals, for union by the first intenlion cannot be contemplated when the ecraseur is used. The favorable result, however, of wounds or operations by this instrument, goes to prove what I have asserted of the bad effects of the effused animal fluids in wounds, either directly on the surface of the wound or by absorption. The ecraseur does not make a rough and ragged wound, as is generally supposed, but the tissues are cut as with scissors, but in such a gradual manner as to give time for the closure of vessels of moderate sizes, and consequently there is no oozing or exuding from its surfaces, and it remains dry, and is followed by no ill-conditioned inflammation. Why is this, when incised wounds are disposed to behave so badly under the same circumstances and in the same hospital? I can see no reason but the effusion of animal juices that takes place in incised wounds, which is prevented by the ecraseur. The incised wound produces less violence, and if everything is favorable, union by the first intention will follow, which cannot be expected of a wound by the ecraseur under the same favorable circumstances. The keen cutting instrument is far preferable in most, if not in all cases, and so far as any absorption of impure matter is dangerous, the effect can be entirely obviated by the application of ice in favorable cases, and if necessary, the application of the alum iron or per-chloride of iron, which arrests at once all oozing of the fluids, and produces a wholesome stimulant effect. These last two applications are only recommended when union by the first intention is not expected, and when there is a general disposition to unhealthy inflammation.

I have been surprised at the readiness with which wounds heal when I have found it necessary to apply these potent styptics, and the uniform healthy character of the reāction after their use.

The ecrasure is better adapted to the removal of hæmorrhoids than anything else; but fifteen minutes to half an hour must be taken for each application, or there will be danger of hæmorrhage. I have been in the habit of using scissors, and applying immediately a sponge of suitable size, saturated with a strong solution of alum iron, which arrests the bleeding with certainty, and the parts give very little trouble afterwards. In a pure state, there is not much difference in effect between the per-chloride and the alum iron, but the per-chloride decomposes readily, gives down a sediment, and leaves an excess of acid, while the alum iron is always neutral.—Whether the French surgeons mean by purulent absorption the actual absorption into the circulation of pus, or mean to express a pyæmic condition, I am unable to determine, tho' it is certain that they do not mean that pus, in its natural state, is absorbed, for it is decided that this cannot take place. They apply it to that pyæmic condition which is attended by great depression, and the formation of matter in different parts of the body, which they attribute to the absorption of impure matter from wounds; it may be disintegrated pus, or other animal fluids. This never takes place in healthy and vigorous subjects, in which healthy exudation of lymph follows wounds, and it is quite plain that its frequency in the Paris hospitals is due, in a great measure, to the want of fresh air and proper nourishment. I speak of the deprivation of fresh air from personal observation, and judge of the diet from the general appearance of the patients. In the London hospitals, surgical patients presenting the appearance of the generality of those in the Paris hospitals, would be ordered beef, mutton, and porter or port wine, etc. This general poverty of the blood prevents adhesive inflamation, and leaves wounds in a condition to absorb the unhealthy fluids that form in them, and the general system in a state to be most seriously affected by it.

By proper preparation, by means of tonics, and proper diet, much may be done to prevent this unfavorable result.—Several years ago, and for several years, operations were disposed to take on a kind of diffusive, unhealthy, cellular inflamation, and in all cases that admitted of delay, I was in the habit of preparing them, by the use of tonics and attention to the stomach and diet, and by these means I almost always avoided this unhealthy process. The late Professor Wedderburn, who was one of the visiting surgeons of the Charity Hospital, during the time referred to, made local applications for the same purposes. His usual application was a strong solution of sulph. quinine, with a slight excess of acid, and he thought it almost a specific. I believe it was of some service, but the more potent applications before mentioned, I am confident, are more sure, if the case should be imminent enough to warrant their use, and I hold that these applications would be warranted in all cases where union by the first intention cannot be expected, and when unhealthy inflamation is probable. These cases, however, are different from the cases of pyæmia in the Paris hospitals, and are probably due more to some epidemic poison and deterioration than to poverty of the blood. Pyæmia has also at times been prevalent, as if some epidemic influence was operating. Tonics, fresh air and proper nutriment constitute the general treatment, and stimulants or potent astringents are proper local applications, and very effective, at least as much so as the ecraseur.

